# A new species of *Arabella* (Annelida, Polychaeta, Oenonidae) from the sea off Gouqi Island, Zhejiang, China

**DOI:** 10.3897/zookeys.1278.182033

**Published:** 2026-04-30

**Authors:** Yehoshafati Elton Anton, Amiri Rajabu Mohamedi, Yongjiu Chen

**Affiliations:** 1 College of Marine Science and Technology, Zhejiang Ocean University, Changzhi Island, Zhoushan, Zhejiang 316022, China College of Marine Science and Technology, Zhejiang Ocean University Zhoushan China

**Keywords:** 16S, *Arabella gouqi* sp. nov., COI, morphology, new record, Oenonidae, taxonomy

## Abstract

*Arabella* is the second-most diverse genus in the family Oenonidae, with about 50 species known so far. However, until now, there has been no detailed taxonomic information on *Arabella* species in the East China Sea. In this study, we describe a new species, *Arabella gouqi***sp. nov**., discovered in the sea off Gouqi Island, Zhejiang, China, using integrated morphological and molecular evidence. This species is characterized by the following features: a pygidium with two swollen pads, short notopodial cirri, a bifid robust left and a gracile falcate right maxilla I (MxI), short left and long right maxilla II (MxII), a posterior postchaetal lobe shorter than the chaetae, and gradually tapering of the ventralmost chaetae. Phylogenetic analysis based on both COI and 16S rDNA gene sequences showed that *A. gouqi***sp. nov**. forms a distinct, fully supported monophyletic clade. These findings were further supported by substantial Kimura 2-parameter (K2P) genetic distances between the new species and other reported *Arabella* species, which ranged from 9.9% to 24.2% for COI and from 5.5% to 25.2% for 16S rDNA. This study provides essential data for understanding the taxonomy of *Arabella* in the East China Sea and emphasizes the need for further research on this group in the region.

## Introduction

*Arabella* Grube, 1850, is the second-most diverse genus in the family Oenonidae, Kinberg, 1865, contributing about 76% of the family species when combined with the genus *Drilonereis* (Zanol et al. 2021; Darbyshire and Kara 2024). Presently, about 50 valid species of *Arabella* have been reported worldwide (Read and Fauchald 2025), distributed from the intertidal zone to the abyssal depths (Steiner and Amaral 2009; Zanol and Ruta 2015; Zanol et al. 2021). The study of this group requires careful observation of the maxillary apparatus, notopodial cirri, the pygidium, and the ventralmost chaeta, as its taxonomy is mainly based on the differences in these structures (Colbath 1989; Zanol and Ruta 2015; Zanol et al. 2021; Darbyshire and Kara 2024).

For a long time, *Arabella* was reported as a cosmopolitan genus; a good example is *Arabella iricolor* Montagu, 1804, originally described from South Milton in the UK. After its original description, the name was further used for other specimens outside of Europe (Imajima and Hartman 1964; Uchida 1968; Fauchald 1970; Rho and Song 1975; Mohammad 1981; Paik 1989; Wang and Huang 1993; Blake et al. 1995; Ribeiro et al. 2018; Darbyshire and Kara 2024; Kang et al. 2024). This is because many earlier taxonomists did not examine crucial diagnostic features such as the maxillary and parapodial structures (Imajima and Hartman 1964; Uchida 1968; Paik 1989; Zanol and Ruta 2015; Darbyshire and Kara 2024; Kang et al. 2024). As a result, the idea that *Arabella* is found all over the world likely stems from inadequate descriptions (Darbyshire and Kara 2024; Kang et al. 2024). The actual diversity in the genus has likely been underestimated (Zanol and Ruta 2015; Ribeiro et al. 2018; Zanol et al. 2021; Darbyshire and Kara 2024). Using new morphological details, along with molecular tools, helps us sort out these species and fill in the molecular data, which are still limited (Zanol et al. 2021; Darbyshire and Kara 2024).

This study presents the first record of a species of the genus *Arabella* collected from the sea off the coast of Gouqi Island, Zhejiang, China, based on morphological and molecular evidence. We utilized the mitochondrial cytochrome *c* oxidase subunit I (COI) gene sequence, which has an appropriate evolutionary rate for characterizing variations among closely related species and geographical populations in various marine organisms, including polychaetes (Park 2018; Kara et al. 2020). To increase the confidence of our description, we also sequenced the mitochondrial 16S ribosomal RNA gene. This work constitutes the first documented occurrence of *Arabella* in the East China Sea, supported by integrated morphological and molecular evidence.

## Materials and methods

### Sample collection and morphological identification

**Figure 1.**
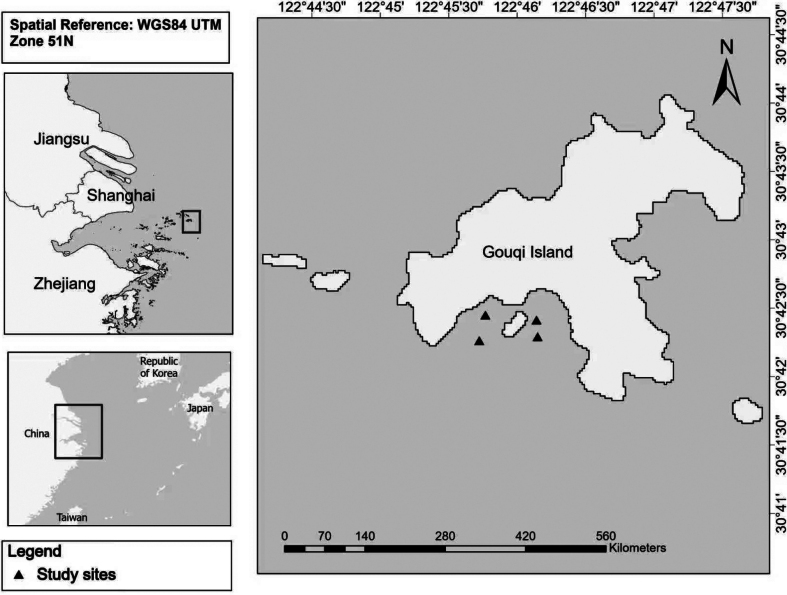
Sampling sites of specimens of *Arabella gouqi* sp. nov. off the coast of Gouqi Island, Zhejiang, China.

A total of eight specimens were collected from four localities (Fig. 1) in mussel farms, specifically from the ropes holding mussels in the sea off Gouqi Island, Zhejiang, China. They were first anesthetized using a 7% magnesium chloride (MgCl_2_) aqueous solution (Darbyshire 2014; Bonyadi-Naeini et al. 2016), then transferred into 90% alcohol for storage, and finally stored at -4 °C for further morphology and molecular identification. During laboratory analysis, body pictures of the specimens were examined using two stereomicroscopes (SZX2-ILLTQ, Olympus, Japan; S9i Leica).

About six specimens were dissected using a thin razor blade under the S9i Leica microscope. When required, we used proteinase K solution to remove tissue and expose internal structures, including maxillae, mandibles, and ventral and dorsal carriers. The targeted body features, including maxillae, mandibles, and chaetae, were mounted on slides with a drop of water, then the slides were observed, and images were captured using both the Olympus SZX2-ILLTQ stereomicroscope and a compound light microscope (Nikon ECLIPSE Ni; China). The nomenclature for maxillary plates and formulae followed the traditional classification of Colbath (1989), Steiner and Amaral (2009), and Zanol et al. (2021). The materials examined in this study were deposited at the Marine Biology Museum of Zhejiang Ocean University in Zhoushan, China.

### Molecular analysis

The genomic DNA was isolated from eight tissue samples preserved in 75% ethanol, following the method described by Aljanabi and Martinez (1997). We used procedures provided by Sangon Biotech (Hangzhou) Co., Ltd, China. PCR was used to amplify both the COI gene and 16S rDNA, using the primer pairs listed in Table 1. PCR runs took place on a C1000 Touch Thermal Cycler with a total volume of about 18.5 μl that contained 12.5 μl of Taq Master Mix (New England Bio Labs), 1 μl of nuclease-free water, 1 μl of forward and reverse primer (10 μM), and 0.8–2.7 μl of template DNA depending on sample concentration.

**Table 1.** The list of primer sets used in this study.

Gene	Primer name	Primer sequence (5’–3’)	Reference
COI	polyLCO	GAYTATWTTCAACAAATCATAAAGATATTGG	Carr et al. 2011
	polyHCO	TAMACTTCWGGGTGACCAAARAATCA	
16S	16SarL	CGCCTGTTTAACAAAAACAT	Palumbi 1996
	16SbrH	CCGGTCTGAACTCAGATCACGT	

The thermal cycling conditions for COI involved 94 °C for 4 min, then 35 cycles of 94 °C for 45 s, 47 °C for 30 s, and 72 °C for 1 min, and finally 7 min at 72 °C. For 16S, thermal cycling conditions started with 95 °C for 3 min, followed by 35 cycles of 94 °C for 1 min, 45 °C for 45 s, and 72 °C for 1 min, and finally 7 min at 72 °C. PCR results were examined by electrophoresis on a 1% agarose gel. The successful samples were then delivered to Sangon Biotech Company in Hangzhou http://www.sangon.com) for bidirectional sequencing. Finally, seven samples for 654 bp COI and four samples for 555 bp 16S rDNA were sequenced successfully.

### DNA data analysis

Preliminary classification was conducted using NCBI nucleotide BLAST and BOLD identification by searching for the most closely related sequences to our specimens (Zhang et al. 2000). Species belonging to the family Eunicidae were used as outgroup taxa, while those in the families Oenonidae and Lumbrineridae were included as ingroup taxa for both COI and 16S phylogenetic analyses; additional *Arabella* species from different localities were analyzed for better comparisons (Table 2). The sequences of both COI and 16S genes were aligned using the ClustalW multiple alignment method (Thompson et al. 1994), then trimmed and edited in BioEdit v. 7.7.1 (Hall 2011). By using both Bayesian Information Criterion (BIC) scores and Akaike Information Criterion (AIC) values, GTR+G was selected as the best-fit substitution model for both COI and 16S (Nei and Kumar 2000). A maximum likelihood (ML) phylogeny was inferred in MEGA X v. 12 (Kumar et al. 2024) with 1000 bootstrap replicates (Felsenstein 1985). The two phylogenetic trees were taken separately into FigTree v. 1.4.4 for visualization and editing (Rambaut 2012). The pairwise interspecific distances for both COI and 16S sequences were computed in MEGA X v. 12.1.0 using the Kimura 2-parameter (K2P) model with 1000 bootstrap replicates (Kimura 1980).

**Table 2.** GenBank and BOLD accession numbers, collection localities, and references for the COI and 16S rDNA sequences used in this study.

Species	COI	16S	Locality	Reference
*Arabella gouqi* sp. nov.	PV548907	–	Gouqi Island, China	This study
	PX495939	PX601586		
	PX495940	–		
	PX495941	–		
	PX495942	PX610608		
	PX495943	–		
	PX495948	PX610609		
	–	PX601587		
*Arabella* sp.	GU362693	GU362681	Qingdao, China	Zhou et al. 2010
*Arabella* sp.	OQ323412	–	Virginia, USA	Direct submission
	OQ323158	–		
	OQ323045	–		
	OQ322696	–		
*Arabella* sp.	–	HM746709	Unknown	Paul et al. 2010
*Arabella* sp.	–	LC787737	Wakayama, Japan	Kobayashi and Abe 2024
*Arabella* sp.	PV563047	–	Nanji Island, China	Direct submission
	PV563048	–		
	PV563051	–		
*Arabella iricolor* Montagu,1804	OR714261	OR725145	South Milton Sands, UK	Darbyshire and Kara 2024
	OR714259	OR725143	Plymouth, UK	
	OR714260	OR725144		
*Arabella semimaculata* Moore, 1911	MK550647	–	California, USA	Direct submission
	MK550651	–	California, USA	
	AY838866	GQ478123	Unknown	Struck et al. 2006
	–	AY838825		
*Arabella umgazanae*	OR714254	OR725147	Mngazana, South Africa	Darbyshire and Kara 2024
	OR714255	OR725148		
	–	OR725149		
	–	OR725150		
*Arabella ampulliformis*	OR714257	OR725146	Lundy Island, UK	Darbyshire and Kara 2024
	OR714258	–	South Milton Sands, UK	
	OR726620	–		
*Arabella protomutans* Orensanz, 1990	OR714256	OR725137	Port San Carlos, Falkland Islands	Darbyshire and Kara 2024
*Arabella* sp.	KANBI1433-19	–	Hawaii, USA	Direct submission
	KANBI759-19	–		
	MW278749	–		
*Drilonereis filum* Claparède, 1868	KT307638	–	North Spain, Spain	Aylagas et al. 2016
	–	OR725138	Northumberland, UK	Darbyshire and Kara 2024
*Drilonereis longa* Webster, 1879	OQ323264	–	Virginia, USA	Direct submission
	OQ323118	–		
	–	AY838828	Unknown	Struck et al. 2006
*Halla okudai* Imajima, 1967	–	LC545402	Fukue Island, Japan	Kobayashi et al. 2020
	LC787733	LC787732	Nagasaki, Japan	Kobayashi and Abe 2024
	LC830815	LC830814	Kanagawa, Japan	Nishi et al. 2024
*Scoletoma laurentiana* Grube, 1863	OR714262	OR725139	South Milton Sands, UK	Darbyshire and Kara 2024
	–	OR725140		
*Oenone fulgida* Lamarck, 1818	AY838872	AY838838	Unknown	Struck et al. 2006
	–	GQ478124		Zanol et al. 2010
*Lumbrineris* sp.	KR916858	–	Faro, Portugal	Lobo et al. 2016
	(BOLD: ACO5551)			
*Marphysa sanguinea* Montagu, 1813	GQ497547	GQ478157	Unknown	Zanol et al. 2010
	–	OR725142	Plymouth, UK	
*Eunice norvegica* Linnaeus, 1767	GQ497541	GQ478147	Unknown	Zanol et al. 2010
	–	OR725141	Dorset, UK	Darbyshire and Kara 2024

## Results

### Systematic accounts

**Class Polychaeta Grube, 1850**

#### Order Eunicida Dales, 1962

**Family Oenonidae Kinberg, 1865**

**Genus *Arabella* Grube, 1850**

##### Arabella gouqi sp. nov.

Taxon classificationAnimaliaEunicidaOenonidae

E5653E6F-E798-557F-B728-CB63D2BABB4A

https://zoobank.org/889762FB-831B-4372-A35D-3D0FAE9B1653

**Figure 2.**
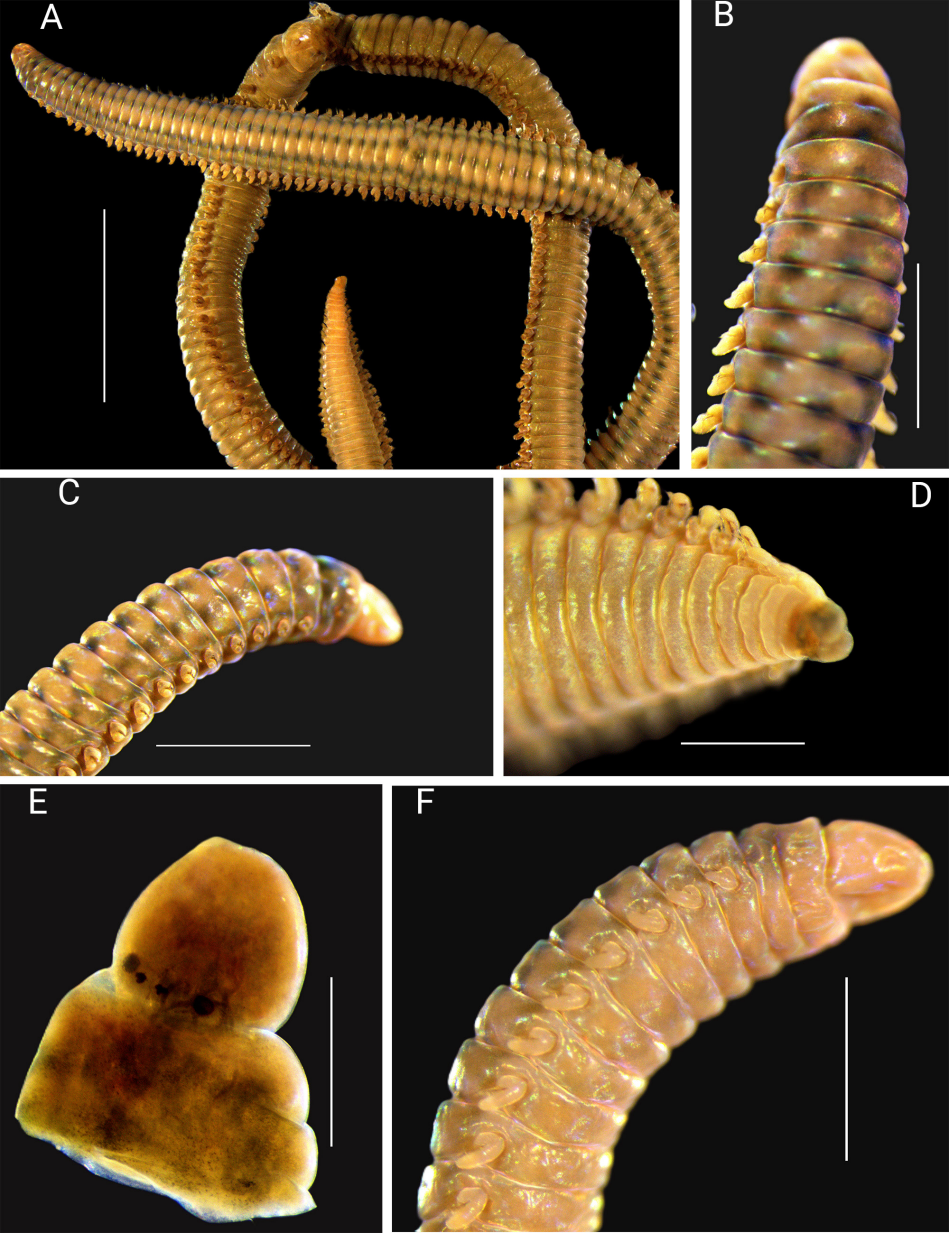
*Arabella gouqi* sp. nov. holotype CMSTT7 (**F**), paratype CMSTT6 (**A**), paratype CMSTT4 (**B**, **E**), paratype CMSTT5 (**C**) and paratype CMSTT1(**D**). **A**. Complete body; **B**. Body, anterior dorsal view; **C**. Anterior, dorsolateral view; **D**. Pygidium ventrolateral view; **E**. Prostomium with four eyes; **F**. Anterior body, ventral lateral view. Scale bars: 7 mm (**A**); 4 mm (**B**–**D**); 12 mm (**E**); 15 mm (**F**).

**Figure 3.**
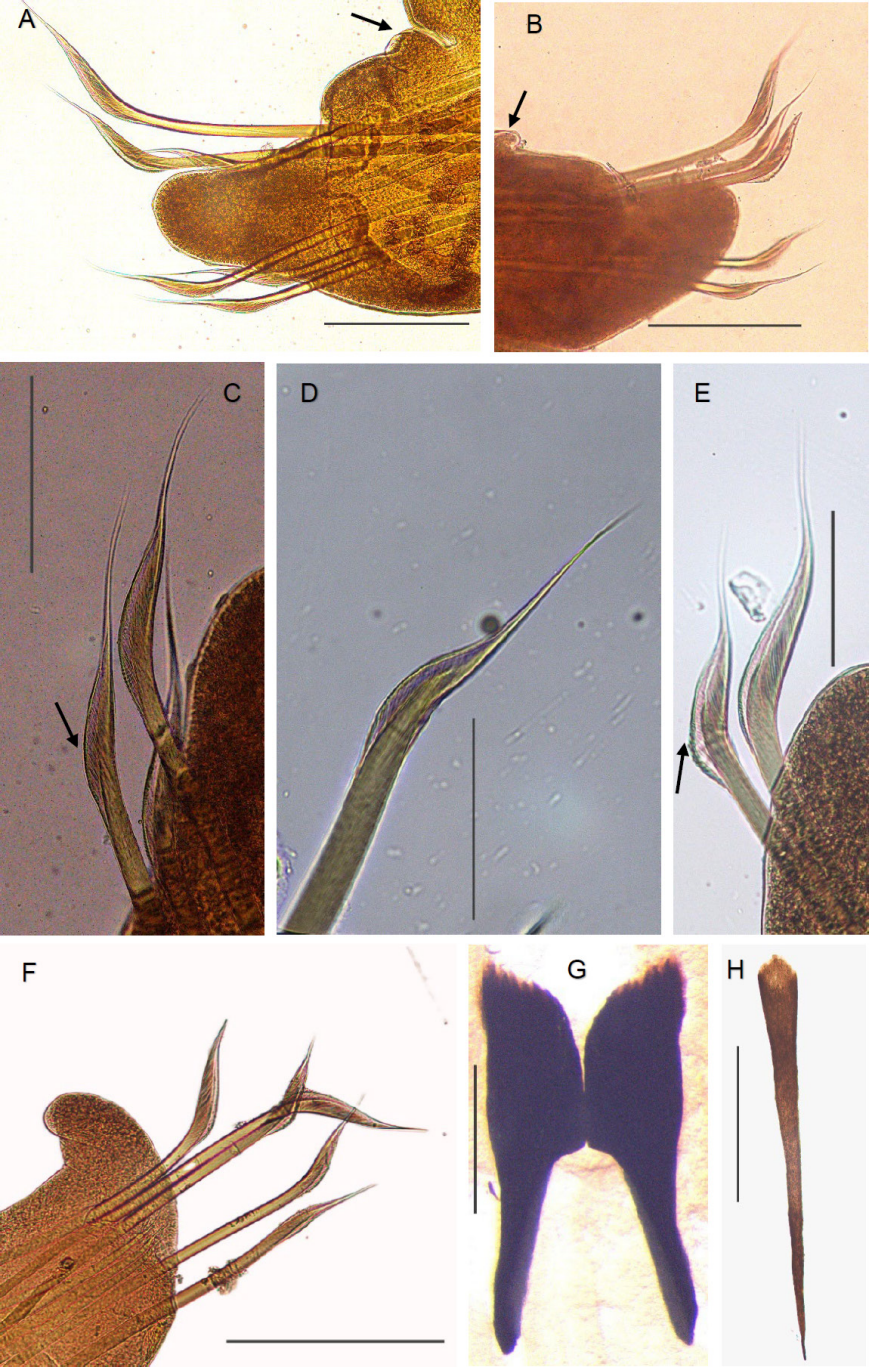
*Arabella gouqi* sp. nov. holotype CMSTT7 (**F**), paratype CMSTT4 (**B**, **D**, **E**), paratype CMSTT5 (**H**), and paratype CMSTT6 (**A**, **C**, **G**). **A**. Left parapodium 22 (arrow pointing to a notopodial cirri); **B**. Right parapodium 24 (arrow pointing to a notopodial cirri); **C**. Right parapodium 22 (arrow pointing to the ventralmost chaetae); **D**. Ventralmost chaetae, right parapodium 23; **E**. Dorsal and mediodorsal chaetae, left parapodium 25 (arrow pointing to coarsely serrated); **F**. Right parapodium 37; **G**. Mandibles; **H**. Ventral carrier. Scale bars: 8 mm (**A**, **B**); 2.8 mm (**C**, **F**); 1 mm (**D**, **E**); 3 mm (**G**, **H**).

**Figure 4.**
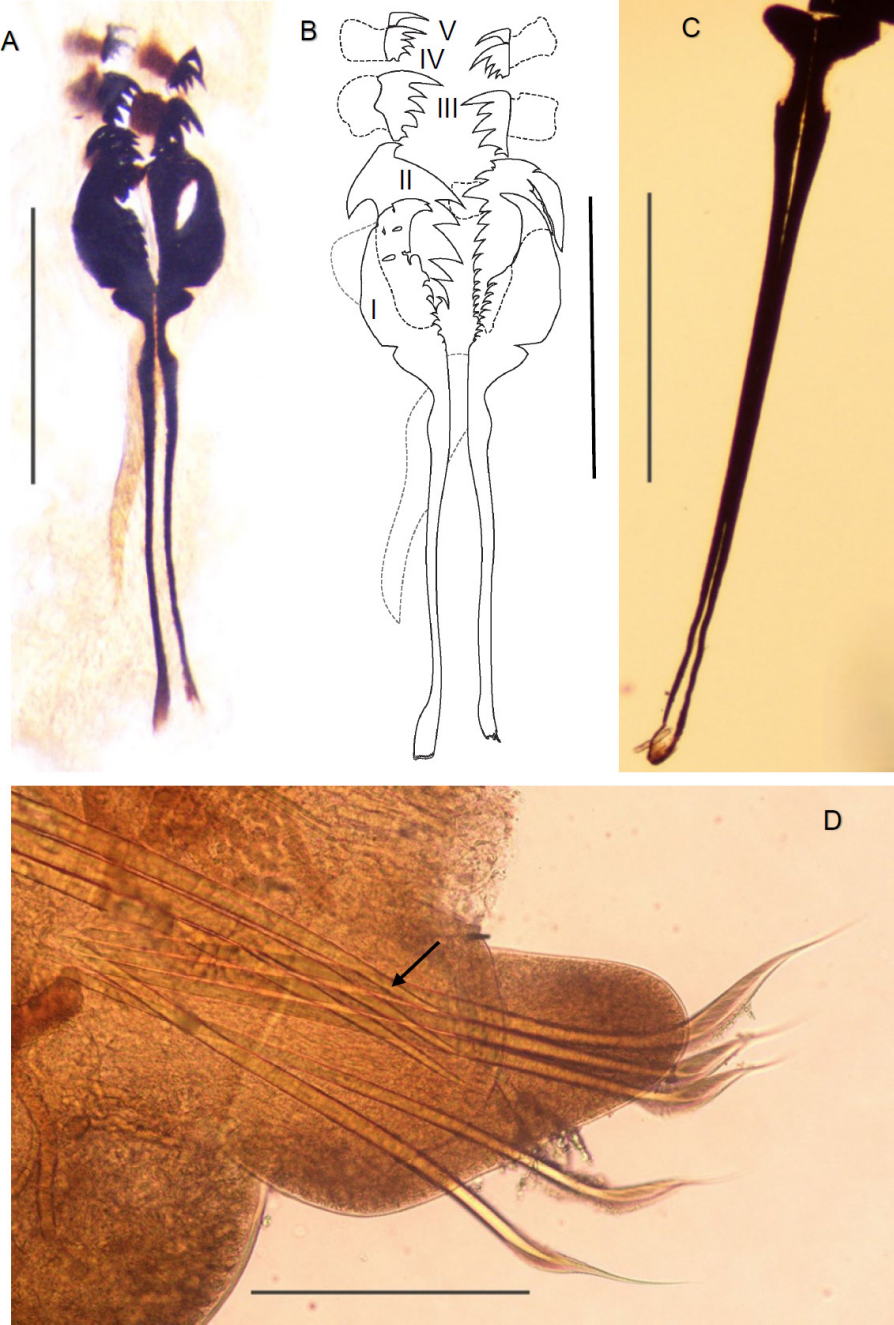
*Arabella gouqi* sp. nov. holotype CMSTT7 (**A**, **B**), paratype CMSTg13 (**C**), and paratype CMSTT4 (**D**). **A**. Maxillae (including ventral and dorsal carriers); **B**. Maxillae I–V; **C**. Dorsal carriers; **D**. Right parapodium 23 (arrow pointing to a notoacicula). Scale bars: 5 mm (**A**, **C**); 4 mm (**B**); 2.5 mm (**D**).

Figs 2A–F, 3A–H, 4A–D

###### Material examined.

***Holotype***. • 1 complete specimen (CMSTT7): China: Zhejiang: Zhoushan; Gouqi Island, 30°42.4546'N, 122°45.7676'E, 20 m depth, 13 Oct 2025, Anton YE leg. ***Paratypes***. 7 specimens: • 1 complete specimen (CMSTg13), 30°42.2679'N, 122°45.7230'E, 1 Dec 2024, other data same as holotype. • 3 complete specimens (CMSTT1, CMSTT2, CMSTT3), 30°42.2963'N, 122°46.1492'E, with other data the same as the holotype. • 3 specimens (CMSTT4, CMSTT5 incomplete, CMSTT6) 30°42.4181'N, 122°46.1410'E; other data same as holotype.

###### GenBank accession numbers.

COI gene: PV548907, PX495939, PX495940, PX495941, PX495942, PX495943, and PX495948. 16S gene: PX601586, PX610608, PX610609, and PX601587.

###### Etymology.

The specific name “*gouqi*” is obtained from the location where the specimens were collected, Gouqi Island, found in Zhejiang Province, China.

###### Diagnosis.

Prostomium tapers anteriorly, with four eyes in a concave arrangement. Peristomium forms double rings. Maxillae with five plate pairs. Shapes both symmetrical and asymmetrical; MxI left bifid robust, right gracile falcate. Long right MxII, short left MxII. MxIII and MxIV with longest anterior-most tooth. MxV one tooth both sides. Maxillary carriers long and slender. Dorsal carriers longer than ventral. Short notopodial cirri. Prechaetal lobe rounded, shorter than chaetae. Posterior postchaetal lobe shorter than chaetae. Chaetae bilimbate, length decreases from median to both dorsal and ventral or dorsal to ventral only. Chaetae coarsely or finely serrated; ventralmost chaetae taper gradually to guards. Pygidium with one pair of distinct swollen pads.

###### Description.

***Holotype***, complete specimen, 58 mm long with 308 chaetigers. Width, 2 mm at chaetiger 10 and 2.5 mm at the central part, including parapodia. ***Paratypes***. 1 incomplete specimen, 103 mm long with 364 chaetigers. Width, 3 mm at chaetiger 10 and 3.2 mm at the central part, including parapodia. 3 complete specimens, 63–125 mm long with 330–524 chaetigers. Width, 1–3 mm at chaetiger 10 and 1.5–3.5 mm at the central part, including parapodia.

Body long, slender, dorsoventrally rounded, dorsal surface more convex. Widest centrally, maintaining width through most of its length, then gradually tapering to the pygidium. Preserved holotype and paratypes pale brown with dark spots forming dorsal lines throughout the body (Fig. 2A–C). Prostomium slightly shorter than peristomium. Posterior deeper than anterior end, dorsally in a slope, ventrally flattened with median groove from posterior to anterior (Fig. 2C, F). Prostomium eyes arranged in a concave shape. Median pair smaller than lateral (Fig. 2E). Peristomium of two distinct rings, both wider than prostomium (Fig. 2B, C, F).

Mandibles black, connected by short ligament, positioned anterior to maxillae when retracted, cutting plates shorter than the mandibular carriers, inner edge shorter than lateral (Fig. 3G). Dorsal maxillary carriers paired widest anteriorly, poorly defined posteriorly; ventral carrier unpaired, widest anteriorly, tapers posteriorly. Ventral carrier is about 2/3 the length of dorsal carriers (Figs 3H, 4A, 4C). Maxillae with five pairs of plates, symmetrical (MxIII, MxIV and MxV) or asymmetrical (MxI and MxII). MxI left bifid robust, right gracile falcate. Long right MxII, short left MXII. MxIII and MxIV with the longest anterior-most tooth, and MxV with one tooth on both sides. Maxillary formula: MxI teeth (2, (6–7)) + (1, (8–9)); MxII teeth (8–9) + (17–18); MxIII teeth 6+6; MxIV teeth 5+5; MxV teeth 1+1. (Fig. 4A, B).

Prechaetal lobe rounded, shorter than chaetae. Posterior postchaetal lobe elongate but shorter than chaetae (Figs 3A, 3B, 4D). Short notopodial cirri present, observed clearly at the anterior and central chaetigers (Fig. 3A, B). 3 notoaciculae present shorter than prechaetal lobe, neuroaciculae not observed (Fig. 4D). 5 to 7 bilimbate chaetae present (the number varies between specimens, but consistent throughout the body), dorsal or mediodorsal chaetae longer than others. Chaetae coarsely serrated (mediodorsal chaetae) or finely serrated (other chaetae), ventralmost chaetae taper gradually to guards (Figs 3A–F, 4D). Chaetal length decreases from median to both dorsal and ventral or dorsal to ventral only (Figs 3A, 3B, 3F, 4D). Pygidium with two distinct swollen pads (Fig. 2D).

###### Distribution.

Currently known only from mussel farms in the sea off the coast of Gouqi Island in Zhejiang, China.

###### Habitat.

Specimens of *A. gouqi* sp. nov. were obtained from the ropes used to hold mussels in mussel farms. The ropes were submerged in seawater to a depth of approximately 20 m.

###### Remarks.

*Arabella gouqi* sp. nov. is characterized by a combination of the following features: a pygidium with two distinct swollen pads; short notopodial cirri; gradually tapering ventralmost chaetae; a bifid robust left MxI; a gracile falcate right MxI; a short left MxII; and a long right MxII.

The gradually tapering ventralmost chaetae have also been reported in *A. iricolor*Montagu 1804, *A. semimaculata* Moore, 1911, *Arabella turbidiricolor* (Kang et al. 2024), *A. protomutans* Orensanz, 1990, *Arabella aracaensis* Steiner & Amaral, 2009, *Arabella pulvinata* Zanol & Ruta, 2015, *Arabella logani* Crossland, 1924, *Arabella longicirrata* Hartmann-Schröder, 1979, *Arabella pectinata* Fauchald, 1970, *A. umgazanae*, and *A. ampulliformis* Darbyshire & Kara, 2024.

However, *A. iricolor*, *A. semimaculata*, *A. aracaensis*, *A. pulvinata*, *A. longicirrata*, and *A. turbidiricolor* are easily distinguished from *A. gouqi* sp. nov. by having a unidentate left MxI (vs. a bidentate left MxI in *A. gouqi* sp. nov.) (Moore 1911; Hartmann-Schröder 1979; Blake et al. 1995; Steiner and Amaral 2009; Zanol and Ruta 2015; Darbyshire and Kara 2024; Kang et al. 2024). Other *Arabella* species reported as having a unidentate left MxI are as follows: *Arabella cincta* Hartmann-Schröder, 1962, from Chile; *Arabella coeca* Fauvel, 1940, from the Adriatic Sea; *Arabella monroi* Colbath, 1989, from Ecuador; *Arabella robusta* Zanol & Ruta, 2015, from Australia; *Arabella atlantica* Crossland, 1924, from the Cape Verde Islands; *Arabella endonata*; and *Arabella longipedata* Monro, 1931, from Australia.

Of the species introduced above, *A. logani* Crossland, 1924, reported from the Gulf of Suez, Egypt, is very close to *A. gouqi* sp. nov. in the shapes of MxI and MxII, the pygidium, the ventralmost chaetae, and the length of the posterior postchaetal lobe in relation to the chaetae. However, the original description by Crossland (1924) shows clearly the presence of five notoaciculae in *A. logani* that differ from *A. gouqi* sp. nov., which has three notoaciculae. Moreover, *A. logani* has 8 chaetae in anterior chaetigers, which outnumber the 5 to 7 chaetae in *A. gouqi* sp. nov. (Darbyshire and Kara 2024). *Arabella umgazanae* Darbyshire & Kara, 2024, (described from Mngazana, South Africa) is closer to *A. gouqi* sp. nov. in having gradually tapering ventralmost chaetae, short notopodial cirri, a bifid left MxI, and a pygidium with one pair of swollen pads. However, the two species can be primarily distinguished by the shape of the pygidial lobes: in *A. umgazanae*, the lobes are semicircular (vs. circular/round lobes in *A. gouqi* sp. nov.). There are also some differences in the number of teeth on MXI, MXII, and MXIV. In addition, in *A. umgazanae*, chaetae decrease in length from dorsal to ventral in all parapodia (vs. chaetal length decreases from median to both dorsal and ventral, or from dorsal to ventral, in *A. gouqi* sp. nov.).

Moreover, *A. protomutans* is similar to *A. gouqi* sp. nov. in the shape of MxI and MxII, the pygidium, the ventralmost chaetae, and the length of the posterior postchaetal lobe in relation to the chaetae. However, the two species differ in the denticulation of maxillae II and III (Orensanz 1990). *Arabella pectinata* Fauchald, 1970, (from Mexico, eastern Pacific) has all teeth evenly long in maxillae III and IV; in addition, each tooth has a short blunt tip; however, *A. gouqi* sp. nov. has alternately long and short teeth, with sharp tips and the longest anterior-most tooth in MxIII and MxIV (Fauchald 1970; Kang et al. 2024). On the other hand, *A. ampulliformis* Darbyshire & Kara, 2024, (reported from the UK) is distinguished from *A. gouqi* sp. nov. in possessing a pygidium with two pygidial lobes extended into short, stout cirri, ampulliform in lateral view (vs. a pygidium with two swollen pads in *A. gouqi* sp. nov.)

Of *A. iricolor* described outside of its original Europe (Montagu 1804) and before redescription by Darbyshire and Kara (2024), including those from America (Colbath 1989), Brazil (Ribeiro et al. 2018), Pakistan (Mustaquim 2000), Australia (Blake et al. 1995; Zanol and Ruta 2015), and Mexico (Fauchald 1970), all were described as having gradually tapering ventralmost chaetae, similar to *A. gouqi* sp. nov. However, with the exception of specimens from Pakistan for which the pygidium shape is unknown, the rest were described with a pygidium bearing at least two cirri (vs. two swollen pads in *A. gouqi* sp. nov.). Other specimens from Caribbean (Treadwell 1921) and Japan (Imajima and Hartman 1964; Uchida 1968) were also described with a pygidium of at least two cirri, which differs from the two swollen pads in *A. gouqi* sp. nov.

Comparisons of *A. gouqi* sp. nov. with other *Arabella* species reported from China, such as *Arabella renierii* Grube, 1877, are challenging due to the lack of detailed morphological descriptions from its original description. Other samples were reported as *A. iricolor* (Wang and Huang 1993; Zhou et al. 2010), but currently no updated morphological features are available for comparison. *Arabella mutans* Chamberlin, 1919, was listed as one of the species found in the East China Sea by Liu (2008). If the material from China resembles the original description, then *Arabella mutans* is easily distinguished from *A. gouqi* sp. nov. in having abruptly tapering ventralmost chaetae and a pygidium with four cirri (vs. gradually tapering ventralmost chaetae and two swollen pads in *A. gouqi* sp. nov.) (Chamberlin 1919; Zanol and Ruta 2015).

*Arabella zonata* Moore, 1903, reported from Japan, can easily be distinguished from *A. gouqi* sp. nov. by having a pygidium with a pair of short lateral cirri (Moore 1903; Hartman 1942) (vs. a pygidium forming two swollen pads with no cirri in *A. gouqi* sp. nov.). Other *Arabella* species, including *Arabella mutans*, Chamberlin, 1919, from Chile, and *Arabella panamensis* Colbath, 1989, from Costa Rica, were all described as having abruptly tapering ventralmost chaetae (Colbath 1989; Steiner and Amaral 2009; Zanol and Ruta 2015). This feature differs from *A. gouqi* sp. nov., which is characterized by gradually tapering ventralmost chaetae. *Arabella iridescens* Treadwell, 1906, reported from Hawaii (Hartman 1942), is easily differentiated from *A. gouqi* sp. nov. through the shape of right MXI and MXII. *Arabella iridescens* has a right MXI roughly triangular in outline (vs. gracile falcate right MXI in *A. gouqi* sp. nov.).

### Phylogenetic analysis and genetic divergence

**Figure 5.**
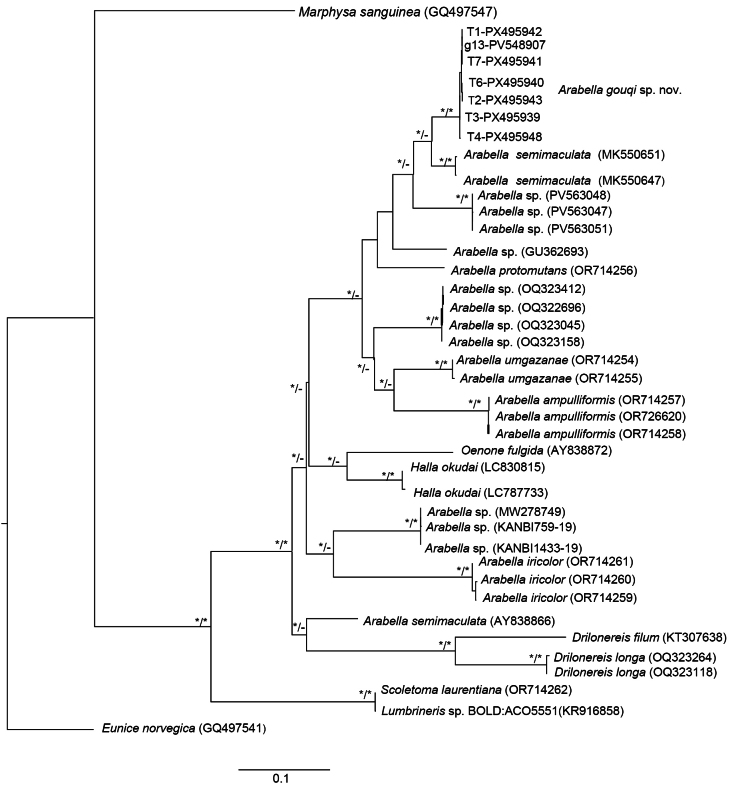
Maximum likelihood (ML) tree based on COI sequences of *Arabella* species from different localities. Asterisks (*/*) at nodes indicate clades with ≥ 95% bootstrap values. The scale bar represents substitutions per site.

**Figure 6.**
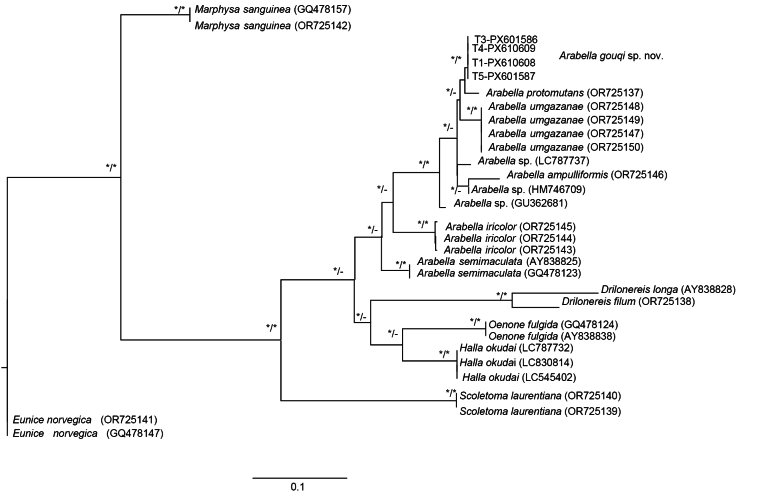
Maximum likelihood (ML) tree based on 16S rDNA sequences of *Arabella* species from different localities. Asterisks (*/*) at nodes indicate clades with ≥ 95% bootstrap values. The scale bar represents substitutions per site.

The maximum likelihood (ML) trees produced from both COI and 16S rRNA sequences revealed *A. gouqi* sp. nov. in a separate monophyletic clade, as supported by 100% and 95% ML bootstrap values, respectively (Figs 5, 6). The K2P distances between *A. gouqi* sp. nov. and other known *Arabella* species ranged from 9.9% to 24.2% for COI and 5.5% to 25.2% for 16S rRNA (Tables 3, 4).

**Table 3.** Genetic divergence (K2P) based on COI between *Arabella* species from different localities.

	Species name	Locality	1	2	3	4	5	6	7	8	9	10
1	*A. gouqi* sp. nov. (PX495941)	Gouqi Island, China										
2	*A. semimaculata* (MK550651)	California, USA	0.099									
3	*Arabella* sp. (PV563047)	Nanji Islands, China	0.145	0.148								
4	*A. protomutans* (OR714256)	Port San Carlos, Falkland Islands	0.177	0.178	0.186							
5	*A. semimaculata* (AY838866)	Unknown	0.217	0.228	0.243	0.202						
6	*Arabella* sp. (OQ323412)	Virginia, USA	0.190	0.196	0.226	0.197	0.216					
7	*Arabella* sp. (GU362693)	Qingdao, China	0.173	0.155	0.168	0.173	0.199	0.219				
8	*A. ampulliformis* (OR714257)	Lundy Island, UK	0.215	0.206	0.251	0.220	0.225	0.208	0.202			
9	*A. iricolor* (OR714260)	Plymouth, UK	0.242	0.258	0.273	0.244	0.244	0.220	0.266	0.291		
10	*Arabella* sp. (KANBI1433-19)	Hawaii, USA	0.223	0.213	0.254	0.272	0.205	0.239	0.239	0.261	0.250	
11	*A. umgazanae* (OR714254)	Mngazana, South Africa	0.194	0.200	0.212	0.174	0.244	0.181	0.187	0.170	0.251	0.253

**Table 4.** Genetic divergence (K2P) based on 16S rDNA between *Arabella* species from different localities.

	Species name	Locality	1	2	3	4	5	6	7	8
1	*A. gouqi* sp. nov. (PX610608)	Gouqi Island, China								
2	*A. semimaculata* (AY838825)	Unknown	0.245							
3	*A. protomutans* (OR725137)	Port San Carlos, Falkland Islands	0.112	0.177						
4	*A. ampulliformis* (OR725146)	Lundy Island, UK	0.133	0.234	0.102					
5	*A. umgazanae* (OR725147)	Mngazana, South Africa	0.068	0.186	0.086	0.116				
6	*A. iricolor* (OR725144)	Plymouth, UK	0.252	0.145	0.201	0.238	0.234			
7	*Arabella* sp. (GU362681)	Qingdao, China	0.110	0.188	0.098	0.131	0.118	0.195		
8	*Arabella* sp. (HM746709)	Unknown	0.107	0.188	0.096	0.100	0.086	0.212	0.108	
9	*Arabella* sp. (LC787737)	Wakayama, Japan	0.055	0.220	0.087	0.087	0.089	0.224	0.104	0.080

Other specimens identified as *A. iricolor* from the Nanji Islands in Zhejiang, China, were likewise placed in a unique monophyletic clade with 100% ML bootstrap support (Fig. 5). The K2P distance based on COI between *Arabella* sp. from Nanji Islands and *A. gouqi* sp. nov. was 14.5% (Table 3), indicating that both species are distinct. A specimen identified as *A. iricolor* from Qingdao in Shandong, China, was found in a distinct clade from *A. gouqi* sp. nov. in both COI and 16S rRNA ML trees (Figs 5, 6). The K2P distance between *Arabella* sp. from Qingdao and *A. gouqi* sp. nov., as determined by COI and 16S rRNA, was 17.3% and 11.0%, respectively (Tables 3, 4). Additionally, the Qingdao specimen was clustered in a separate clade with specimens from the Nanji Islands (Fig. 5), with a 16.8% K2P distance based on COI sequences (Table 3); these species were most likely misidentified.

Specimens reported as *A. iricolor* from China (Nanji Islands and Qingdao), the US (Virginia and Hawaii), and the UK (South Milton Sands and Plymouth) were all clustered in different clades in both COI and 16S rRNA ML trees (Figs 5, 6). The overall mean K2P distances between them were 20.0% and 16.0%, respectively (Tables 3, 4). However, specimens from South Milton Sands and Plymouth (UK) were clustered together with 100% and 98% ML bootstrap support in the COI and 16S rDNA ML trees, respectively (Figs 5, 6), indicating the occurrence of *A. iricolor* in the UK. The COI and 16S rDNA sequences of specimens from South Milton Sands and Plymouth (UK) showed K2P distances of 0.72% and 0.57%, respectively (Tables 3, 4). Other specimens reported as *A. iricolor* from Virginia and Hawaii were in separate clades (Fig. 5), with a K2P distance of 23.9% based on COI sequences (Table 3), indicating that they are distinct species.

*Arabella semimaculata* Moore, 1911, reported from California, USA, formed a unique monophyletic clade with a 99.9% ML bootstrap support (Fig. 5). Another *A. semimaculata* with GenBank accession number AY838866 (from an unknown location) proved to be in a different clade from the specimens derived from California, USA (Fig. 5). Their K2P distance based on COI was 22.9% (Table 3); thus, they are separate species. On the 16S rDNA phylogenetic tree, *A. semimaculata* with GenBank accession numbers AY838838 and GQ478124 were clustered together with 99% ML bootstrap support. These specimens may have been collected in the same region. There are no 16S rDNA sequences available for *A. semimaculata* from California, USA; therefore, comparisons were impossible.

Another *Arabella* species reported from Mngazana, South Africa, *A. umgazanae* Darbyshire & Kara, 2024, was clustered together in COI and 16S rRNA ML trees, with 100% bootstrap support (Figs 5, 6). *Arabella ampulliformis* Darbyshire & Kara, 2024, reported from the UK, formed a distinct monophyletic clade, supported by 100% bootstrap support on the COI tree.

## Discussion

The combined updated morphological and molecular analyses provide strong evidence that the specimens collected from the sea off Gouqi Island represent a new species, *A. gouqi* sp. nov. This study constitutes the first documented occurrence of the genus *Arabella* in the East China Sea, supported by detailed morphological and molecular evidence.

Morphologically, *A. gouqi* sp. nov. is characterized by a combination of a pygidium with two distinct swollen pads; short notopodial cirri; gradually tapering ventralmost chaetae; a bifid robust left MxI and a gracile falcate right MxI; a short left MxII and a long right MxII. As suggested by Colbath (1989), Zanol and Ruta (2015), Zanol et al. (2021), Darbyshire and Kara (2024), and Kang et al. (2024), the provided combination of features is crucial and enough to differentiate *A. gouqi* sp. nov. from its closely related species.

Other *Arabella* species, including *A. mutans* Chamberlin, 1919, found in the East China Sea, were listed by Liu (2008); however, no detailed morphological support is available. In addition, Zhou et al. (2010) and other records available in GenBank reported the occurrence of *A. iricolor* from Qingdao and Nanji Islands, respectively. Apparently, *A. gouqi* sp. nov. is genetically distinct from all these species. This highlights unresolved taxonomic complexity in this region and requires more careful redescription of all previously reported *Arabella* species in this and nearby regions.

The present study also reveals a more localized distribution of *Arabella*, as recently pointed out by Darbyshire and Kara (2024) in the redescription of *A. iricolor* Montagu, 1804, and Kang et al. (2024). Our study supports that all specimens previously identified as *A. iricolor* should be revised before using this name, because, apart from the specimens reported in the UK, other specimens reported as *A. iricolor* from other places, including China (Nanji Islands and Qingdao) and the US (Virginia and Hawaii), were all phylogenetically reciprocally separate.

## Conclusions

This study describes a new species of *Arabella* using integrated morphological and molecular data. It constitutes the first documented occurrence of the genus *Arabella* in the East China Sea based upon morphological and molecular evidence. To characterize this new species, we used updated morphological traits, including the maxillary apparatus, ventralmost chaetae, notopodial cirri, and pygidium. This study provides essential data for understanding the taxonomy of *Arabella* in the East China Sea and emphasizes the need for further research on this group in the region.

## Supplementary Material

XML Treatment for Arabella gouqi

## Additional information

### Artificial Intelligence (AI) use

The authors accept full responsibility for the content of the manuscript, including the disclosure of any use of AI.

No AI tools were used in the preparation of this manuscript.

### Author ORCIDs

Y.E. Anton https://orcid.org/0009-0003-0143-6260

A.R. Mohamedi https://orcid.org/0009-0000-3747-1242

Y. Chen https://orcid.org/0000-0002-1591-3190

### Data availability

All of the data that support the findings of this study are available in the main text or Supplementary Information.

## Supplementary materials

Supplementary material 1 Sample collection sites Data type xlsx Explanation note The location where specimens were colleted, at the Sea off the coast of Gouqi Island, Zhejiang, China. https://binary.pensoft.net/file/1614637This dataset is made available under the Open Database License (http://opendatacommons.org/licenses/odbl/1.0/). The Open Database License (ODbL) is a license agreement intended to allow users to freely share, modify, and use this Dataset while maintaining this same freedom for others, provided that the original source and author(s) are credited. Yehoshafati Elton Anton, Amiri Rajabu Mohamedi, Yongjiu Chen

Supplementary material 2 Newick Export for CO1 ML phylogenetic tree Data type nwk Explanation note Export file used for contruction of CO1 ML phylogenetic tree. https://binary.pensoft.net/file/1614638This dataset is made available under the Open Database License (http://opendatacommons.org/licenses/odbl/1.0/). The Open Database License (ODbL) is a license agreement intended to allow users to freely share, modify, and use this Dataset while maintaining this same freedom for others, provided that the original source and author(s) are credited. Yehoshafati Elton Anton, Amiri Rajabu Mohamedi, Yongjiu Chen

Supplementary material 3 Newick Export for 16S ML phylogenetic tree Data type nwk Explanation note Newick Export file for 16S ML phylogenetic tree. https://binary.pensoft.net/file/1614639This dataset is made available under the Open Database License (http://opendatacommons.org/licenses/odbl/1.0/). The Open Database License (ODbL) is a license agreement intended to allow users to freely share, modify, and use this Dataset while maintaining this same freedom for others, provided that the original source and author(s) are credited. Yehoshafati Elton Anton, Amiri Rajabu Mohamedi, Yongjiu Chen
